# Whole genome sequencing of measles viruses: the benefit for outbreak investigation, The Netherlands, 2013–2014

**DOI:** 10.1007/s11262-026-02253-8

**Published:** 2026-06-19

**Authors:** Rogier Bodewes, Cyril Maissan, Linda van de Nes-Reijnen, Marjan Boter, Susanne C. van den Boom, Florian Zwagemaker, Susan Hahné, Tom Woudenberg, Wilhelmina L. M. Ruijs, Marion P. G. Koopmans, Chantal B. E. M. Reusken, Rory D. de Vries, Richard Molenkamp, Rik L. de Swart, B. Oude Munnink

**Affiliations:** 1 Centre for Infectious Disease Control, National Institute for Public Health and the Environment (RIVM), Bilthoven, The Netherlands; 2 Department of Viroscience, Erasmus MC, University Medical Center Rotterdam, Rotterdam, The Netherlands; 3 Infectious Diseases and Immunology Division, Virology Section, Department of Biomolecular Health Sciences, Faculty of Veterinary Medicine, Utrecht University, Utrecht, The Netherlands

**Keywords:** Measles virus, Sequencing, Molecular surveillance, Genetic variation

## Abstract

Measles is a highly contagious infectious disease and remains a major cause of global morbidity and mortality. Molecular surveillance of measles virus (MV) is used for tracing transmission chains, by, for instance, distinguishing between repeated introductions and endemic circulation. WHO guidelines used in the global measles surveillance network recommend sequencing of a 450-nucleotide region at the C-terminal end of the N-gene (N450). Given the limited genetic variability of MV, whole-genome sequencing would increase the resolution and provide a more detailed molecular surveillance of circulating MV. In this study, we describe an amplicon-based nanopore sequencing protocol for generating near complete whole MV genome sequences (nWGS; 15,813 nucleotides). We applied this protocol to samples collected during an epidemic of measles in the Netherlands in 2013–2014, involving an estimated 30,000 cases, to quantify the sequence variation of nWGS during this epidemic. While this was considered an epidemic with very limited sequence variation based on analysis of the N450 region, different molecular clusters were identified by phylogenetic analysis of nWGS genomes. Notably, the number of single nucleotide variants (SNVs) between viruses detected during the early and late phase of the epidemic varied among molecular clusters. Four epidemiological clusters could be identified in the beginning of the epidemic, of which 2 were supported by molecular data. Analysis of nWGS genomes suggested that the epidemic started with either a single unnoticed introduction into the Netherlands and a few unnoticed generations or with at least 2 introductions at the same time, the latter option is supported by epidemiological data. These results can aid in interpreting MV sequence variation during transmission chains, outbreaks and epidemics in countries or regions approaching measles elimination.

**Supplementary Information:**

The online version contains supplementary material available at 10.1007/s11262-026-02253-8.

## Introduction

The recent global surge in measles cases underscores its status as a highly contagious infectious disease and a major cause of morbidity and mortality in children and young adults [1–3]. The causative agent, measles virus (MV), is an enveloped virus with a single-stranded negative-sense RNA genome of typically 15,894 nucleotides and belongs to the family Paramyxoviridae, genus *Morbillivirus*. Partial sequencing of MV genomes was shown to support molecular epidemiology, identifying 24 genotypes of which only two have been detected after 2020 (B3 and D8) [4]. The MV genome is relatively stable compared to other RNA viruses, with an estimated 3.9 × 10^−4^ substitutions per site per year [5].

Molecular surveillance is a valuable tool for tracing MV transmission chains, which can in combination with epidemiological data distinguish between independent introductions and endemic circulation. Following WHO guidelines, molecular surveillance is based on sequencing of a 450-nucleotide region at the C-terminal end of the MV N-gene (N450). Given the limited genetic variability in N450 sequences of currently circulating MV strains, increased resolution is needed for a more accurate interpretation of molecular data as part of outbreak analytics and more granular tracking of transmission chains. Multiple new approaches have been developed in recent years, including protocols for (near) whole-genome sequencing (nWGS) [6–11]. However, our understanding of nucleotide diversity within measles outbreaks or epidemics remains limited. Such knowledge is crucial for countries or regions that are approaching measles elimination and who aim to use whole-genome sequencing to determine whether multiple epidemiological clusters are the result from separate introductions or from a single transmission chain [12].

Between May 2013 and March 2014, a large measles epidemic occurred in the Netherlands mainly among a socio-geographically clustered population with low vaccination coverage [13]. A total of 2700 laboratory-confirmed or epidemiologically linked cases were reported; however, due to substantial underreporting, the true number of cases was estimated to exceed 30,000 [14]. The epidemiology of this epidemic was described previously [13]. In brief, in the first months of 2013 sporadic import cases and small limited outbreaks were reported in various regions in the Netherlands. On May 27, 2013, two outbreaks were reported in two different regions with a low vaccination coverage: an outbreak at a household in Alblasserdam and an outbreak at a primary school close to Brakel. In the following weeks, cases were reported in multiple regions with a low vaccination coverage. The peak of the epidemic was in the second week of July with 180 reported cases. There was a subsequent decline in the number of reported cases during summer school holidays, followed by another peak in October. Thereafter, the number of cases declined until the last case of the epidemic was reported in March 2014.

During this epidemic, N450 sequences were obtained from 509 cases with a MV RT-qPCR-positive sample. The epidemic strain was a MV genotype D8 strain, MVs/Alblasserdam.NLD/22.13, identical to named strain MVs/Taunton.GBR/27.12 (distinct sequence identifier; DSID 2595) [13]. In addition, minimal sequence variation was observed in the N450 sequences obtained during the epidemic.

The aims of this study were to develop an amplicon-based nanopore nWGS protocol and use this protocol to quantify MV sequence variation during the measles epidemic in 2013–2014. In addition, nWGS data were interpreted with limited available epidemiological data, in order to gain insights into the origin of this epidemic.

## Materials and methods

### Samples and metadata

Between May 27, 2013 and March 12, 2014, 2766 measles cases were reported [13]. It was considered an epidemic based on the sudden increase in the number of reported measles cases above what is normally expected in a specific population and area. The N450 sequence was obtained for the previous study from samples from 509 cases that tested positive by MV RT-qPCR [15] during the reported epidemic [13]. From the total of 509 cases, samples from 87 cases were selected for nWGS. Selected samples had been collected during the early (weeks 22–25 of 2013) or late (weeks 2–5 of 2014) stage of the epidemic to study viral evolution. Additional samples collected from cases during the epidemic were included based on availability, time and location (Table S1, Figure S1). Preferably, samples from different time points and locations during the epidemic were used to evaluate the sequence diversity. To evaluate possible transmission of MV in the Netherlands before the first cases were reported that were considered the start of the epidemic, nWGS genomes were also obtained from viruses detected in 5 cases that were reported in 2013 in the Netherlands before the start of the epidemic. None of these cases had an epidemiological link to cases belonging to the epidemic. Available metadata included sample type (throat swab, oral fluid, urine, or liquor), place of residence, age, date of sample collection, and/or onset of exanthema. From a small number of cases during the early phase of the epidemic, the name of the school, or epidemiological links with other cases were known. After the early phase of the epidemic, the outbreak response shifted from focusing on identifying and reporting cases to limit the epidemic to mainly focusing on protecting vulnerable individuals against severe disease. From a few cases, the place of residence could not be determined and hence, these viruses were named MVs/NLD/ww.yy instead of MVs/city or province.NLD/ww.yy. Molecular surveillance of MV is part of the Public Health Act (BWBR0024705) in the Netherlands. Therefore, no ethical approval and informed consent were required when using pseudonymized routine surveillance data.

### Amplicon-based nanopore sequencing

Amplicon-based nanopore sequencing was performed using an in-house amplicon-based nanopore sequencing approach, resembling a protocol that was described previously [7]. Sequencing was performed at two different laboratories, RIVM and Erasmus MC, with slightly different protocols. Primers were designed and optimized for MV genotypes B3 and D8 to obtain 39 partially overlapping amplicons covering 15,813 nucleotides of the genome (Table S2). During pilot studies, (near) complete MV sequences were also obtained for MV strains belonging to other genotypes. In brief, nucleic acids were extracted from clinical specimens using the MagNApure 96 (MP96) with the total nucleic acid kit small volume (Roche) or the High-Pure RNA isolation kit (Roche). Complementary (c)DNA was prepared using random hexamers (50 µM), dNTP mix (10 mM), Superscript IV-buffer (5x), 100 mM DTT and Superscript IV Reverse Transcriptase (200 u/µl) (Invitrogen). MV amplicons were obtained in 4 different primer-pools, with primers in different quantities (Table S2). Those were used in the MV-amplicon-based PCR (98 °C 30 s, 98 °C 15 s; 64 or 65 °C; 2 or 5 min 47x) together with the Q5 Hot Start High-Fidelity Polymerase (New England Biolabs). After PCR, MV amplicon pools were mixed for each sample with ratios (25:25:7:7) or (11:11:3:3). PCR amplicons were quantified using the Qubit 4 Fluorometer with the Qubit 1 × dsDNA HS assay kit (Invitrogen), and after end repair using the NEBNext Ultra II End Prep reagents (New England Biolabs). Native barcode ligation was performed after which amplicons were purified using AMPure XP beads (Beckman Coulter), pooled and sequenced using R10.4.1 flow cells (Oxford Nanopore Technologies), according to manufacturer’s recommendations. Consensus sequences (> 50 × depth of coverage) were generated using the ViroConstrictor pipeline [16]. In each run, a negative and, at RIVM, a positive control (MV genotype C2) was included. Regions with a coverage of less than 50 were filled with Ns. A complete genome was obtained in every run for the positive control. Validation of the nWGS Nanopore protocol was performed via 2 methods: (1) comparison with > 100 N450 sequences obtained by Sanger sequencing, (2) comparison of consensus sequences of > 20 specimens repeated ≥ 2 in different runs and different tenfold dilutions of 2 measles virus genotype B3 and D8 isolates (1:100 until 1:100,000, corresponding to estimated Ct values of 23 until 33 or 25 until 35, respectively). Consensus sequences were submitted to GenBank, accession numbers PX475853–PX475941 and PX979535–PX979543. Raw sequence data were submitted to the European Nucleotide Archive, study accessions PRJEB106934 and PRJEB107067. Sequences were included in the study if they had ≥ 90% genome coverage. From samples included in the study at RIVM, 90% had a coverage of at least 50 reads for > 90% of the genome. In total, 69 sequences were included that were determined at RIVM, while 23 sequences were included determined at Erasmus MC.

### Comparison of nWGS MV sequences

All sequences included in the analysis were aligned and the range, average and median of the number of single nucleotide variants (SNVs) between viruses of the 2013–2014 epidemic in the Netherlands were calculated in MEGA-X [17]. For comparison of viruses detected early and late during the epidemic, viruses were selected that were collected from patients with onset of exanthema in weeks 22–25 of 2013 (*n* = 18; early) and weeks 2–5 of 2014 (*n* = 7; late) (Table S1).

### Phylogenetic analysis

In the manuscript describing the epidemiology of the 2013–2014 measles epidemic, only the results of the molecular typing of the outbreak strain (MVs/Alblasserdam.NLD/22.13) were presented [13]. Therefore, a comparison of phylogenetic analysis based on N450 and nWGS included in this study and closely related sequences was done. First, an alignment of the nWGS genomes and several closely related MVs was prepared in MEGA-X. A phylogenetic tree of the obtained nWGS genomes was constructed using IQ-TREE2 with the maximum likelihood method and the predicted best-fit substitution model TPM2u + F according to Bayesian information criterion (BIC) using the ModelFinder function [18–20]. The ultrafast bootstrap approach was used to calculate the branch support with 1,000 bootstrap alignments, and bootstrap values ≥ 95 were considered significant [21]. Subsequently, sequences were trimmed to the N450 region and a phylogenetic tree was prepared with the HKY + F substitution model according to BIC.

In order to provide more insights in the origin of this epidemic, a time-resolved phylogenetic analysis was performed using BEAST version 1.10.4 with a strict molecular clock and a Bayesian SkyGrid Tree prior. For this analysis, nWGS genomes were selected that were collected from patients before the start of the epidemic, during the beginning of the epidemic (date of onset of disease between 22 May and 1 July) and several closely related strains available on GenBank. The Markov chain Monte Carlo (MCMC) was run for 50,000,000 states with a sampling frequency of 1 every 1000 states, resulting in effective sample sizes (ESS) of at least 300 for all model parameters according to analysis with Tracer v1.7.1. TreeAnnotator v1.10.4 was used to build a maximum clade credibility (MCC) tree with median node heights and a 10% burn-in. Trees were visualized using FigTree v1.4.4. To study possible options for the origin of the epidemic, the model of Penedos and colleagues was used as described previously to predict whether viruses that were detected early during the epidemic were epidemiologically related [12]. A substitution rate of 5.6 × 10^−4^ substitutions per site per year was used and a number of 15,813 sites. The substitution rate was obtained by BEAST analysis of the 87 nWGS genomes that were reported during the measles epidemic in 2013–2014.

## Results

### Validation of the amplicon-based sequencing method

Comparison of > 100 N450 sequences (both samples from this study and samples from recent cases) obtained by Sanger sequencing and nWGS nanopore sequencing resulted in identical sequences. In addition, comparison of consensus sequences of more than 20 specimens repeated 2 or more times in different runs and dilutions resulted in identical sequences, although there was an increasing number of regions with dropouts and ambiguous bases with higher dilutions. Furthermore, comparison of 10 specimens sequenced at both RIVM and Erasmus MC resulted in identical sequences. From the samples selected from the epidemic for this study, all 87 had a coverage of ≥ 90% and were included in the analysis. From these 87 nWGS genomes, 64 were complete, 10 had a coverage ≥ 98 and < 100%, 10 had a coverage ≥ 95 and < 98%, and 3 had a coverage between ≥ 90 and < 95%. Regions of the genome with a coverage of less than 50 reads (filled with Ns for the analysis) for > 10 nWGS genomes were regions between nucleotide positions 5258 and 5504, 6053 and 6411, and 7626 and 7888, respectively, amplicons 14, 16, and 20.

### Analysis of N450 sequences

From the 87 MV nWGS sequences included in the present study and that were considered part of the epidemic, 79 had N450 sequences that were identical to MV genotype D8 named strain MVs/Taunton.GBR/27.12 (DSID 2595, GenBank accession number JX984461). Furthermore, 8 viruses had 1 SNV in the N450 compared to MVs/Taunton.GBR/27.12, of which 3 (MVs/Dodewaard.NLD/29.13, MVs/Dodewaard.NLD/30.13, MVs/Gelderland.NLD/31.13) at the same position (Figure S2). The N450 sequence of these 8 viruses had no exact match with viruses present in the GenBank database. One out of 8 viruses (MVs/Zuid-Holland.NLD/49.13/4) had an exact match with several viruses present in the MeaNS2 database.

### Analysis of nWGS genomes

The mean number of SNVs between the nWGS sequences included in the study that were part of the epidemic was 6 (Table 1), ranging from 0 to 19; 19 SNVs were present between MVs/Vianen.NLD/3.14 and MVs/Zuid-Holland.NLD/49.13/4 (Table S3). Late MV nWGS genomes were more divergent than early sequences. The median number of SNVs of all viruses that were reported early during the epidemic was 2 (range 0–9); 9 SNVs were detected between MVs/Zuid-Holland.NLD/23.13 and MVs/Leerbroek.NLD/25.13 or MVs/Utrecht.NLD/23.13. The median number of SNVs of all viruses that were reported late during the epidemic was 9, with a maximum of 19 SNVs between MVs/Vianen.NLD/3.14 and MVs/Nieuw-Lekkerland.NLD/2.14. When comparing genomes detected early during the epidemic with genomes detected late, the median number of SNVs was 7. Only 2 SNVs were detected between 8 identical nWGS genomes (MVs/Brakel.NLD/22.13/2, MVs/Brakel.NLD/22.13/3, MVs/Gelderland.NLD/23.13, MVs/Gelderland.NLD/23.13/3, MVs/Gelderland.NLD/25.13, MVs/Waarder.NLD/23.13, or MVs/Zeeland.NLD/22.13) and MVs/NLD/4.14, while 18 SNVs were detected between MVs/Zuid-Holland.NLD/23.13 and MVs/Vianen.NLD/3.14.

**Table 1:** Overview of results of SNV analysis between nWGS genomes included in this study that were part of the outbreak. The number of sequences included in each analysis is included in the second row

Number of SNV	All^1^	Early^2^ and late^3^	Early	Late	Early vs late
Median	6	4	2	9	7
Mean	6	5	3	10	7
Maximum	19	19	9	19	18
Minimum	0	0	0	0	2

^1^nWGS genome collected from patients during the reported outbreak in 2013–2014 (*n* = 87)

^2^nWGS genomes collected from patients with onset of exanthema in weeks 22–25 of 2013 (*n* = 18)

^3^nWGS genomes collected from patients with onset of exanthema in weeks 2–5 of 2014 (*n* = 7)

### Analysis of epidemiological data before and in the early phase of the epidemic

**Fig. 1:**
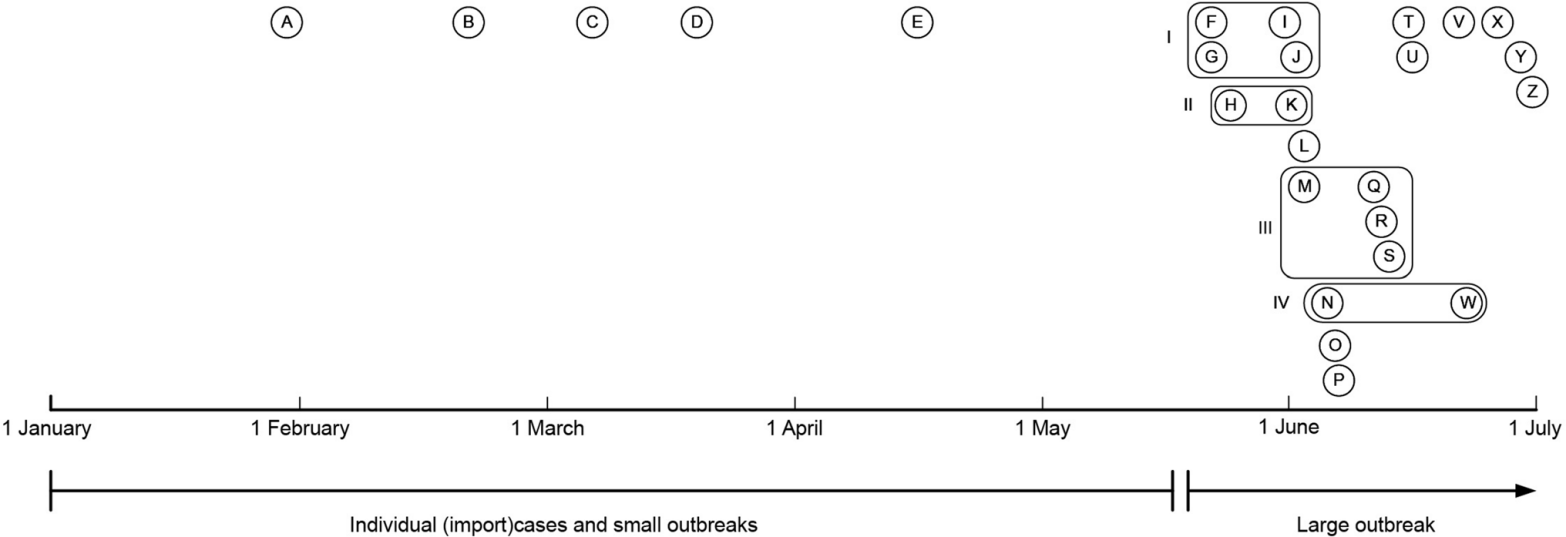
Overview of measles cases from which nWGS genomes were obtained before and in the beginning of the epidemic. Individual cases are indicated with letters (A–Z). Location on the Y-axis is based on onset of disease. Known epidemiological clusters are indicated with Latin numbers (I–IV) and lines around the cases that belong to the epidemiological cluster

Measles cases reported in 2013 before the first reported cases of the epidemic were either sporadic cases or small outbreaks related to a known import case. During the first weeks of the epidemic, 4 epidemiological clusters (epi-cluster) could be defined consisting of 2 to 4 cases (Fig. 1). Epi-cluster I consisted of 4 patients that were either family members or attended the same school. Epi-cluster II consisted of two patients with both an epidemiological link to a school (other school than epi-cluster I). Epi-cluster I was in Alblasserdam (33 km from Wijk en Aalburg and 30 km from Brakel), while the school of epi-cluster II was in Wijk en Aalburg. Epi-cluster I and II were reported on the same day; there was no epidemiological link between these clusters. Epi-cluster III consisted of 4 reported patients, of which 3 patients attended the same school and 1 patient was infected by another patient that attended that school. Epi-cluster IV consisted of 2 patients who both attended the same school. All identified epi-clusters occurred at different schools.

### Comparison of results of epidemiological analysis and molecular surveillance

**Fig. 2:**
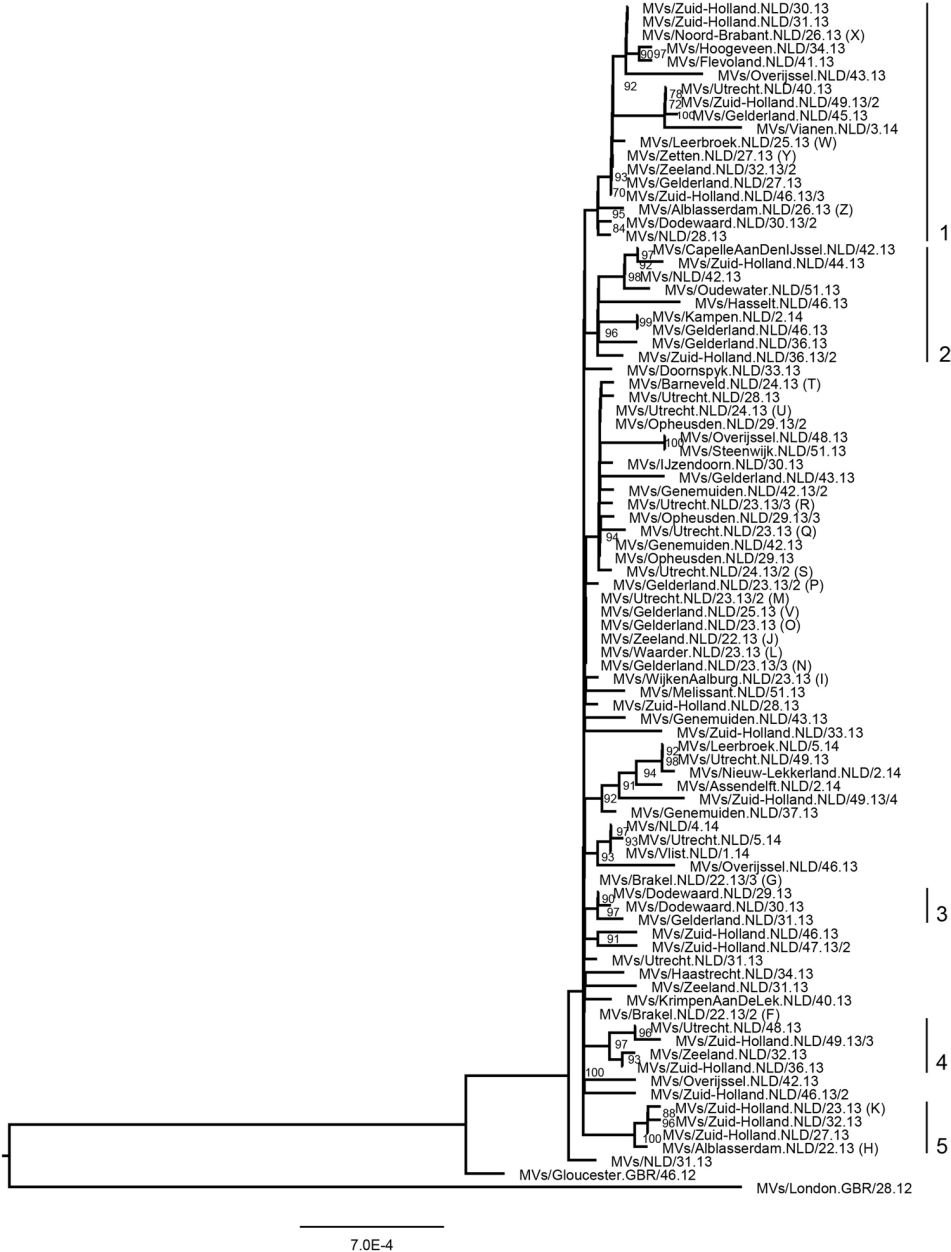
Phylogenetic analysis of nWGS genomes detected during the 2013–2014 epidemic in the Netherlands. Phylogenetic tree was prepared with the maximum likelihood method and the TPM2u + F substitution model and 1000 bootstrap replicates. Molecular clusters with significant bootstrap support (≥ 95) are indicated with numbers; only bootstrap values ≥ 70 are indicated. Letters behind the virus names correspond to case numbers indicated in Fig. 1. MVs/Gloucester.GBR/46.12 and MVs/London.GBR/28.12 were included as reference strains (both 15,678 nucleotides)

Phylogenetic analysis based on nWGS genomes resulted in 5 molecular clusters with significant bootstrap support (Fig. 2). Only 2 of the 5 defined molecular clusters contained sequences from early cases associated with epi-clusters. Molecular cluster 5 with cases K and H was part of epi-cluster II; there were 3 SNV between MVs/Alblasserdam.NLD/22.13 and MVs/Zuid-Holland.NLD/23.13. Patients W (epi-cluster IV), X, Y, and Z were part of molecular cluster 1, while there was no epidemiological link identified between these cases. MVs/Gelderland.NLD/23.13/2 (patient N) and MVs/Leerbroek.NLD/25.13 (patient W) were part of epi-cluster IV. Three SNV were detected between these 2 viruses and these nWGS genomes were not part of the same molecular cluster.

For the other epi-clusters the genomic resolution of the nWGS genomes was too limited to support the epidemiological data. Three nWGS genomes from epi-cluster I (MV/Brakel.NLD/22.13/2, MVs/Brakel.NLD/22.13/3, MVs/Zeeland.NLD/22.13) were identical while there was 1 SNV detected with MVs/WijkenAalburg.NLD/23.13 (Figs. 1, 2). There were maximum 3 SNV detected between nWGS genomes collected from measles cases (M, Q, R, S) of epi-cluster III. Multiple nWGS were determined from other cases reported in the beginning of the epidemic that were (nearly) identical but there was no known epidemiological link between these cases.

### Analysis of the origin of the epidemic

**Fig. 3:**
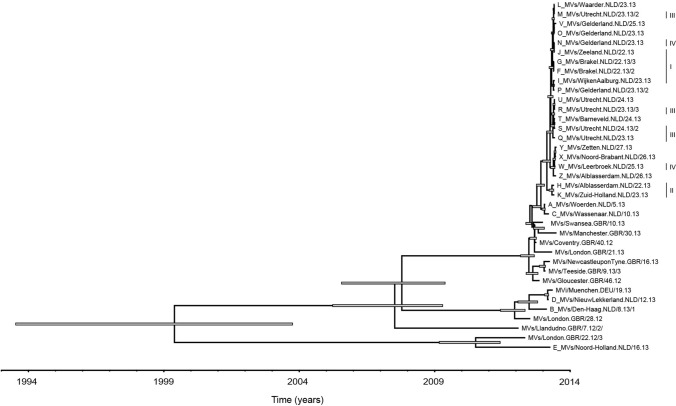
Time-measured phylogenetic analysis of nWGS genomes. nWGS genomes included in the analysis were detected before and in the beginning of the epidemic in the Netherlands. In addition, several closely related nWGS genomes available on GenBank were included. Letters before the virus names correspond to case letters in Fig. 1, while epidemiological clusters are indicated with I–IV. Gray bars indicate 95% HPD

Measles viruses MVs/Den-Haag.NLD/8.13/1 (measles case B), MVs/NieuwLekkerland.NLD/12.13 (measles case D) and MVs/Noord-Holland.NLD/16.13 (measles case E) were all measles genotype D8 viruses but were genetically distinct from viruses detected during the epidemic (Fig. 3). Measles viruses MVs/Woerden.NLD/5.13 (measles case A), MVs/Wassenaar.NLD/10.13 (measles case C) and MV from the beginning of the epidemic (cases F to Z) were estimated to have a common ancestor prior (September 2012 and January 2013) to the onset of the epidemic. MV detected during the beginning of the epidemic (MV from cases F to Z) were estimated to have a common ancestor between December 2012 and April 2013, also prior to the epidemic. MV collected from measles cases H and K (molecular cluster 5, epi-cluster II) were estimated to have a common ancestor between March and May 2013, while also MV collected from all other measles cases that were part of the early phase of the epidemic were estimated to have a common ancestor between February 2013 and May 2013. These results suggest that epi-cluster I and II do not share a direct common ancestor.

Comparison of two very early nWGS genomes of epi-cluster I and II (cases F and H; MVs/Brakel.NLD/22.13/2 and MVs/Alblasserdam.NLD/22.13) indicated that there were 5 SNV between both viruses, while there were 0 SNV between two other very early sequences of epi-cluster II (cases F and G; MVs/Brakel.NLD/22.13/2 and MVs/Brakel.NLD/22.13/3). Application of the model described by Penedos and colleagues indicated that it was unlikely (with a Poisson interval, *α*, of 0.99) that MVs/Brakel.NLD/22.13/2 and MVs/Alblasserdam.NLD/22.13 shared a direct common ancestor, which confirms the results of the time-measured phylogenetic analysis.

Comparison with available nWGS from other countries indicated that nWGS genomes from patients during the epidemic in the Netherlands were genetically most similar to viruses as MVs/Swansea.GBR/10.13 (GenBank accession number KT732247) and MVs/Manchester.GBR/30.13 (GenBank accession number KT732261). However, the estimated time to the most recent common ancestor was between August and November 2012 for these viruses and viruses collected from cases A–Z.

## Discussion

In the present study, nWGS was applied to a proportion of samples collected during an epidemic of MV in the Netherlands in 2013–2014. We observed differences in SNV variation between MV genomes detected early and late during the epidemic. Analysis of nWGS genomes increased the molecular resolution compared to N450, and 5 molecular clusters could be identified. Analysis of nWGS genomes supported 2 epidemiological clusters (I and II), but the molecular resolution was too low to confirm all separate epidemiologically linked transmission chains. For example, cases of epidemiological cluster II belonged to molecular cluster 5, while nWGS genomes from cases of epi-cluster I did not form a separate molecular cluster, but 3 nWGS genomes were identical and one differed by only 1 nucleotide. On the other hand, cases W, X, Y, and Z were part of molecular cluster 1, but there was no epidemiological link reported between these cases.

For most samples we obtained 15,813 nucleotides of the MV genome, whereas the typical genome size of MV is 15,894 nucleotides. The exact definition of nWGS MV genomes is unclear. In the WHO laboratory network MV sequences lacking only the 5′ and 3′ termini are entitled WGS without termini (WGS-t; 15,678 nucleotides). We propose defining nWGS as genomes with a size between 15,678 and 15,894 nucleotides. A limitation of this study is that from 23 of the 87 genomes included from the measles 2013–2014 epidemic less than 15,813 nucleotides could be obtained. This decreased the molecular resolution compared to analysis of complete genomes. However, only 3 nWGS genomes were filled with Ns in the non-coding region between the M and F gene, with a relatively high genetic variation.

Another limitation from this study is the relatively limited availability of epidemiological data to combine it with the nWGS genomes. Only 2700 cases were reported (of which 888 were laboratory confirmed and the N450 sequence was obtained from 509 cases) while the estimated size of the epidemic was more than 30,000 cases [14]. Ideally, interpretation of MV sequence variation along a transmission chain should be based on genetic variability data obtained from well-investigated transmission chains confirmed by both epidemiological contact tracing and WGS. This knowledge from multiple studies can then be used to demonstrate molecular clusters and (im)probable transmission chains when there are no epidemiological links between cases. However, combined WGS and epidemiological data was only available from a few clusters in the beginning of the epidemic. Thereafter, there was rapid spread of MV to multiple parts in the country and the focus on the public health response shifted to protecting vulnerable individuals against severe disease. In addition, measles cases were reported at holiday locations in July and August in the Netherlands, with possible mixing of genetic variants when children were going to school again. This made it difficult to use schools as an epidemiological link after the summer holidays. From those few small well-investigated transmission chains, limited sequence variation was observed ranging from 0 to 3 SNV, which is similar to previous studies [11, 22].

Existing epidemiological data indicated that the epidemic started at two places at the same time, one at a family in Alblasserdam epi-cluster I) and one at a school in Wijk en Aalburg (epi-cluster II). The presence of these 2 epi-clusters in the beginning of the epidemic was supported by analysis of nWGS genomes. Viruses from these 2 clusters had a possible shared common ancestor in the first months of 2013. Viruses detected in the beginning of 2013 in the Netherlands had a possible shared common ancestor in the last months of 2012 or January 2013. The origin of the most common recent ancestor of MVs/Brakel.NLD/22.13/2 (case F; epi-cluster I) and MVs/Alblasserdam.NLD/22.13 (case H, epi-cluster II) might be an earlier undetected introduction in the Netherlands and a few missed generations or there were two distinct introductions from (a) different country or countries. A similar situation occurred during other measles outbreaks [8, 11, 22]. These options stay open, also since there is only limited nWGS data available from other countries from that period. Furthermore, we cannot exclude that there were more introductions from (nearly) identical viruses during the epidemic. However, the available epidemiological data indicated that all reported cases from this epidemic were infected in the Netherlands. Of interest, the nWGS genome of MVs/NLD/31.13 was genetically distinct from other viruses collected from patients during the epidemic, although the N450 was identical to MVs/Alblasserdam.NLD/22.13. Unfortunately, very limited metadata was available from this case.

In conclusion, results of our study suggest that nWGS could be a useful tool for identification and surveillance of MV transmission chains in countries or regions that are in the elimination phase of measles. Additional studies focusing on smaller clusters (e.g., at a school or family) are needed to understand the possible variation of MV nWGS in single transmission chains.

## Supplementary Information

Below is the link to the electronic supplementary material.

Supplementary file1 (DOCX 516 KB)

Supplementary file2 (XLSX 85 KB)

## Data Availability

Consensus sequences were submitted to GenBank, accession numbers PX475853–PX475941, PX979535–PX979543. Raw sequence data were submitted to the European Nucleotide Archive, study accessions PRJEB106934 and PRJEB107067.
