# Supplementary material for: Whole genome sequencing of measles viruses: the benefit for outbreak investigation, The Netherlands, 2013–2014
Source: Virus Genes. 2026 Jun 19;62(4):548–56. doi: 10.1007/s11262-026-02253-8 (PMC13461698; doi:10.1007/s11262-026-02253-8)
Supplement: Supplementary file 1 [file 11262_2026_2253_MOESM1_ESM.docx]

**Supplementary figures**

Supplementary figure 1

**Figure S1**. Map of the Netherlands with locations of residence (based on 4 digital postcode number) of measles cases from which nWGS gemomes were included in this study. In black squares are locations of residence indicated from measles patients that were reported before the start of the epidemic. In white, light grey and dark grey are locations of residence indicated from measles patients that were reported during the epidemic in the respectively early, middle and late phase.

Supplementary figure 2

**Figure S2**. Phylogenetic tree of N450 sequences collected from measles viruses detected during the 2013-2014 epidemic in the Netherlands and closely related sequences based on WGS. The phylogenetic tree was prepared using the HKY+F model according to BIC with 1000 bootstrap replicates. No bootstrap values >70 were calculated.
